# Adult-Onset IgA Vasculitis Presenting as an Unusual Rash and Pancolitis

**DOI:** 10.7759/cureus.26311

**Published:** 2022-06-24

**Authors:** Eunhae Yeo, Bradley D Kaptur, Nicholas J Peterman, Rukhsaar Khanam, Tsungyen Chen

**Affiliations:** 1 Medicine, Carle Foundation Hospital, Urbana, USA; 2 College of Medicine, Carle Illinois College of Medicine, Champaign, USA; 3 Internal Medicine, Carle Foundation Hospital, Urbana, USA

**Keywords:** rheumatology & autoimmune diseases, high dose corticosteroids, pancolitis, rash cutaneous lesions, henoch-schönlein purpura (iga vasculitis), adult iga vasculitis

## Abstract

A 47-year-old female presented with complaints of abdominal pain and a history of new-onset maculopapular rash. A workup including laboratory and imaging studies, colonoscopy, and biopsy was performed that led to the diagnosis of adult-onset IgA vasculitis. The patient responded well to intravenous methylprednisolone and was followed up as an outpatient where she continued with oral methylprednisolone and azathioprine. This case is noteworthy for the unusual adult-onset presentation with primarily gastrointestinal symptoms and atypical rash pattern. Furthermore, while very effective in this patient, the use of corticosteroids is a treatment decision that has some controversy in the current literature.

## Introduction

Immunoglobulin A (IgA) vasculitis, previously called Henoch-Schonlein purpura, is an immune complex-mediated small-vessel vasculitis. It is typically characterized by palpable purpura, arthritis, and abdominal pain [1]. The disease typically affects children, with presentation in adults being less common [2]. Due to the poor characterization of adult IgA vasculitis, the reported incidences are varied: some report values as low as 1/1,000,000 in the population, while other groups cite an incidence of 5.1/100,000 people [3,4].

The onset of IgA vasculitis is often idiopathic. However, there have been reports in the literature of a variety of potential triggers including foods, medications, bacterial and viral infections, insect bites, and immunizations [5-7]. More recently, there have been reports of adult IgA vasculitis in both the context of coronavirus disease 2019 (COVID-19) infection and COVID-19 vaccination [8].

The presentation and course of IgA vasculitis differ between adults and children. Adults tend to have a higher frequency of persisting and relapsing disease, with renal involvement being the main determinant of chronic disease activity [4]. While GI involvement of the disease in adults is not unusual (estimated prevalence of 37% to 65%), it is much less common for GI manifestations to be present at the onset of the disease (estimated prevalence of 14%) [9]. When GI symptoms do present, they are typically abdominal pain, diarrhea, nausea, and vomiting (in order of decreasing frequency) [10]. It has previously been suggested that adult patients with GI bleeding tend to have worse renal outcomes [11]. Previous works have suggested that GI or renal involvement in the disease may be predicted by smoking status, the distribution of skin lesions, and the neutrophil-to-lymphocyte ratio [12].

Treatment of the condition has been typically focused on symptomatic relief, due to the benign course of the disease [13]. However, the treatment of severe disease, characterized by severe GI complications or proliferative glomerulonephritis, has been more controversial in the literature [13]. Some have suggested that glucocorticoids are useful in the management of these severe manifestations; however, several studies have shown no benefit over placebo in various measured outcomes [14]. Rituximab has shown some promise in a few studies as well [15,16].

In this report, we present a case of adult-onset IgA vasculitis with an atypical presentation of primarily GI symptoms and an atypical rash, which was managed with corticosteroids.

## Case presentation

**Figure 1:**
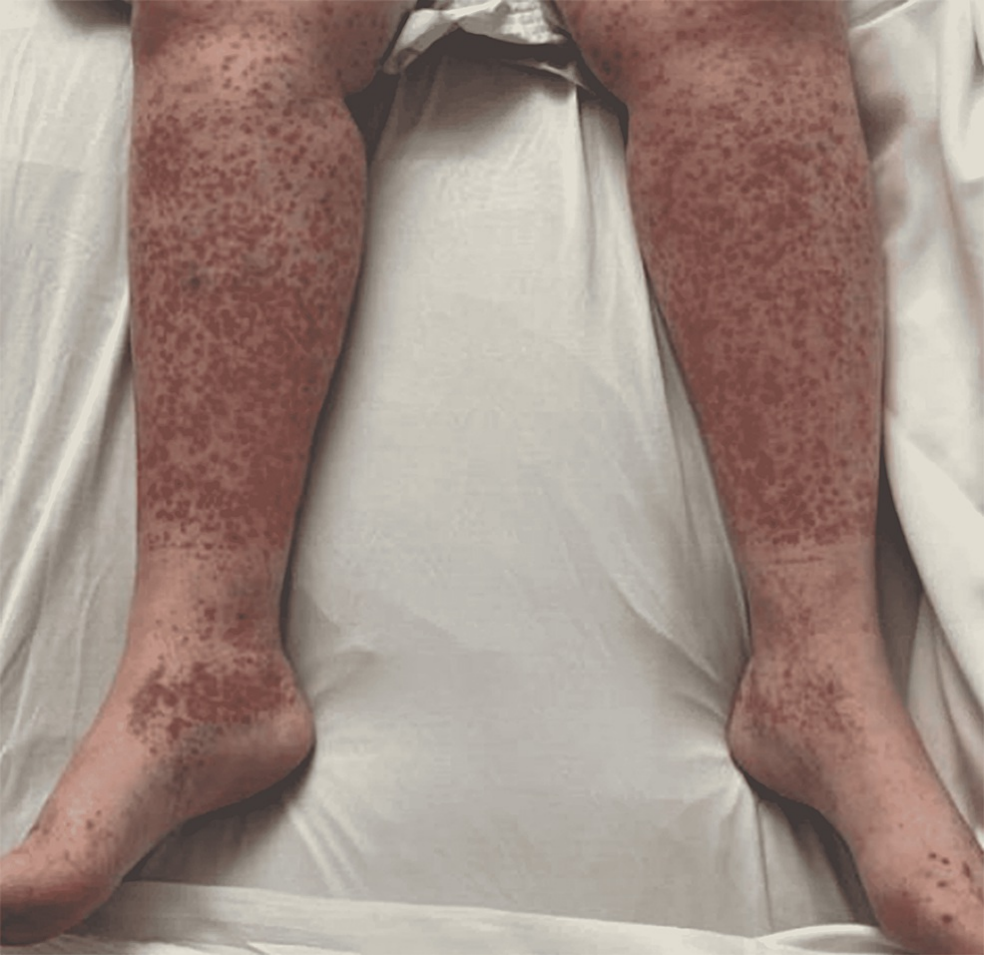
Bilateral maculopapular rash on our patient’s lower extremities observed during her hospital stay.

A 47-year-old female presented to our emergency department with a chief complaint of abdominal pain that had begun a day prior to this encounter. She did not have regular medical follow-up and had no significant past medical or surgical history. She reported that one week before this episode began, she attended an outdoor birthday party. After the party, she started to notice a diffuse bilateral maculopapular rash that began at her sock line (Figure 1). The day immediately prior to arrival at our hospital, she had presented to an urgent care clinic, where she was given an injection of prednisone and prescribed oral prednisone 20 mg twice daily. After she started taking the steroid, she developed significant abdominal pain, vomiting, and profuse watery diarrhea.

On presentation, her abdominal pain was diffuse and non-radiating; her abdomen was tender to palpation. She had severe nausea, vomiting, and loose stools. She denied any hematuria, myalgia, or arthralgia. Her initial laboratory findings are presented in Table 1. Her aspartate transaminase (AST), alanine transaminase (ALT), and lipase were within the normal range. Her electrolytes were unremarkable. Urinalysis was grossly negative and demonstrated no significant proteinuria.

**Table 1:** Patient's laboratory values on presentation. CRP: C-reactive protein; BUN: blood urea nitrogen

Laboratory Test (Normal Range)	Patient’s Values
White blood cell count (4,500-11,000/mm^3^)	17,700/mm^3^
Absolute neutrophils (2,500-6,000/mm^3^)	14,800/mm^3^
Absolute lymphocytes (1,000-4,800/mm^3^)	1,700/mm^3^
CRP (< 10 mg/L)	7.8 mg/L
BUN (6-24 mg/dL)	23 mg/dL
Creatinine (0.7-1.3 mg/dL)	1.09 units/L

An autoimmune workup was performed that showed negative antinuclear antibody (ANA), antineutrophil cytoplasmic antibodies (ANCA), rheumatoid factor, anti-dsDNA, anti-SCL 70, anti-Jo, anti-tTG, and celiac serology. Complement, cryoglobulins, IgG, IgA, and angiotensin-converting enzyme (ACE) levels were all found to be within normal limits. An infectious disease workup showed negative HIV antigen antibody, hepatitis acute panel, rapid plasma reagin (RPR), lyme serology, *Clostridioides difficile* toxin, and GI pathogen panel.

**Figure 2:**
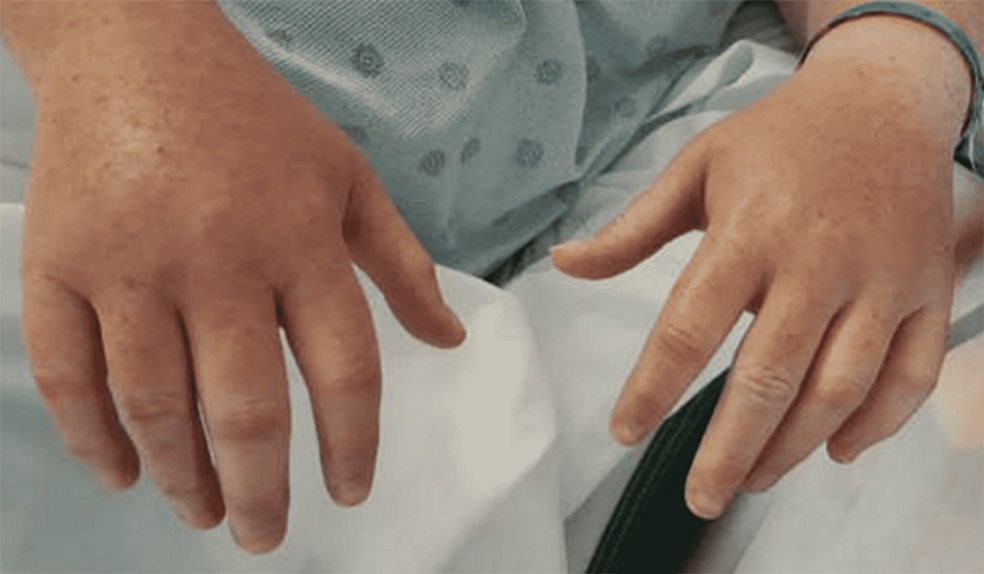
Marked swelling of the patient’s right hand, with some swelling of the left hand. Maculopapular rash is present bilaterally.

During her hospital course, the rash began to resolve on her shins but spread up to her abdomen and upper extremities. On the third day of her hospital stay, she developed right-sided swelling of the face that began on her right eyelid. It then continued to spread, covering her right cheek, lips, and neck. She had marked swelling of the right hand, with minimal swelling of the left (Figure 2). There was no tongue swelling; however, she did report an increased difficulty in swallowing. The swelling was slightly tender to palpation but not pruritic. CT scans of her neck and chest with venogram did not reveal thrombosis or extravascular compressive mass and ruled out superior vena cava syndrome.

**Figure 3:**
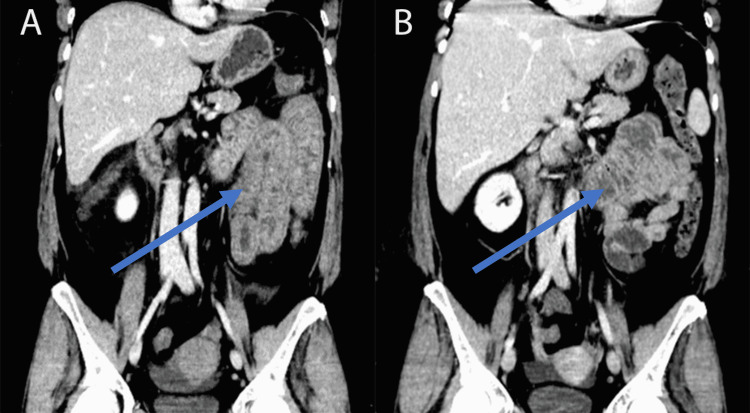
CT abdomen and pelvis with contrast (A) on day of admission and (B) nine days later, showing some improvement of pancolitis (blue arrow).

**Figure 4:**
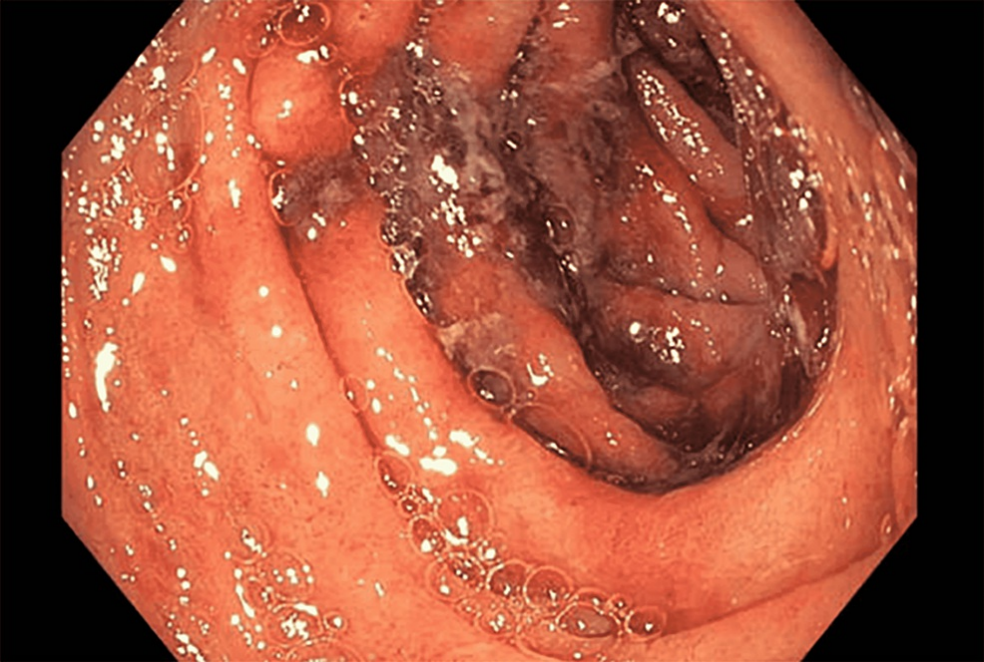
Upper endoscopy (EGD) of the second portion of duodenum with segmental severe inflammation with hemorrhage characterized by adherent blood, congestion, edema, erosions, erythema, friability, and deep ulcerations. EGD: esophagogastroduodenoscopy

**Figure 5:**
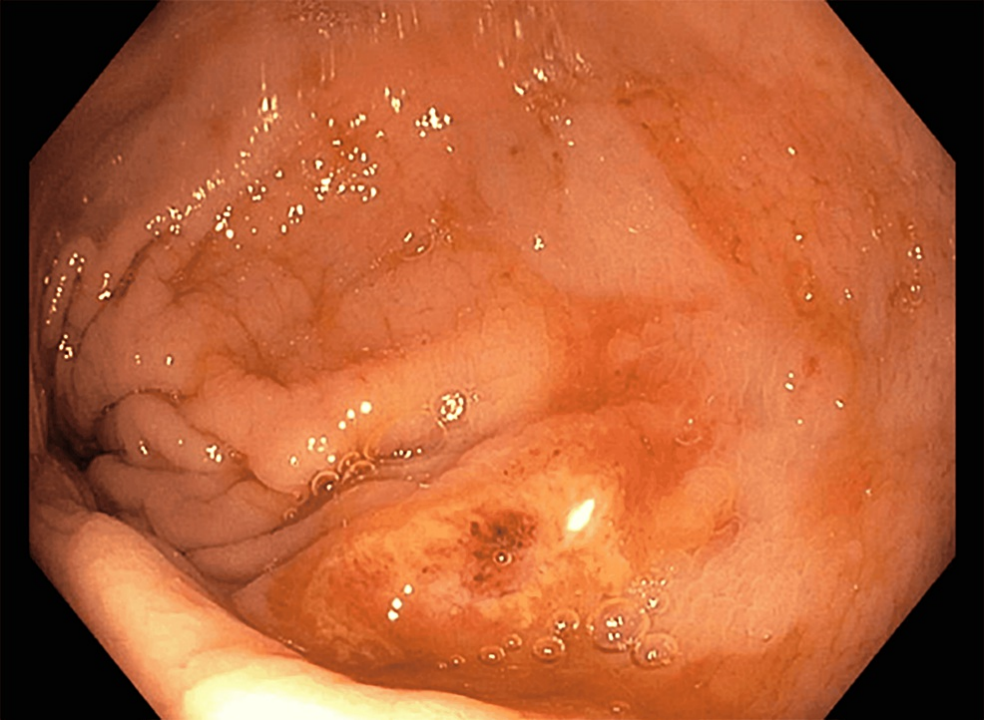
Colonoscopy demonstrating patchy moderate inflammation characterized by congestion, edema, erosions, and erythema found in the rectum.

A CT scan of her abdomen and pelvis was performed and noted inflammation throughout small and large intestines and small volume ascites (Figures 3A, 3B). Subsequently, an upper GI endoscopy was performed and found severe duodenitis with hemorrhage (Figure 4), and a colonoscopy was significant for ileitis and proctitis (Figure 5).

**Figure 6:**
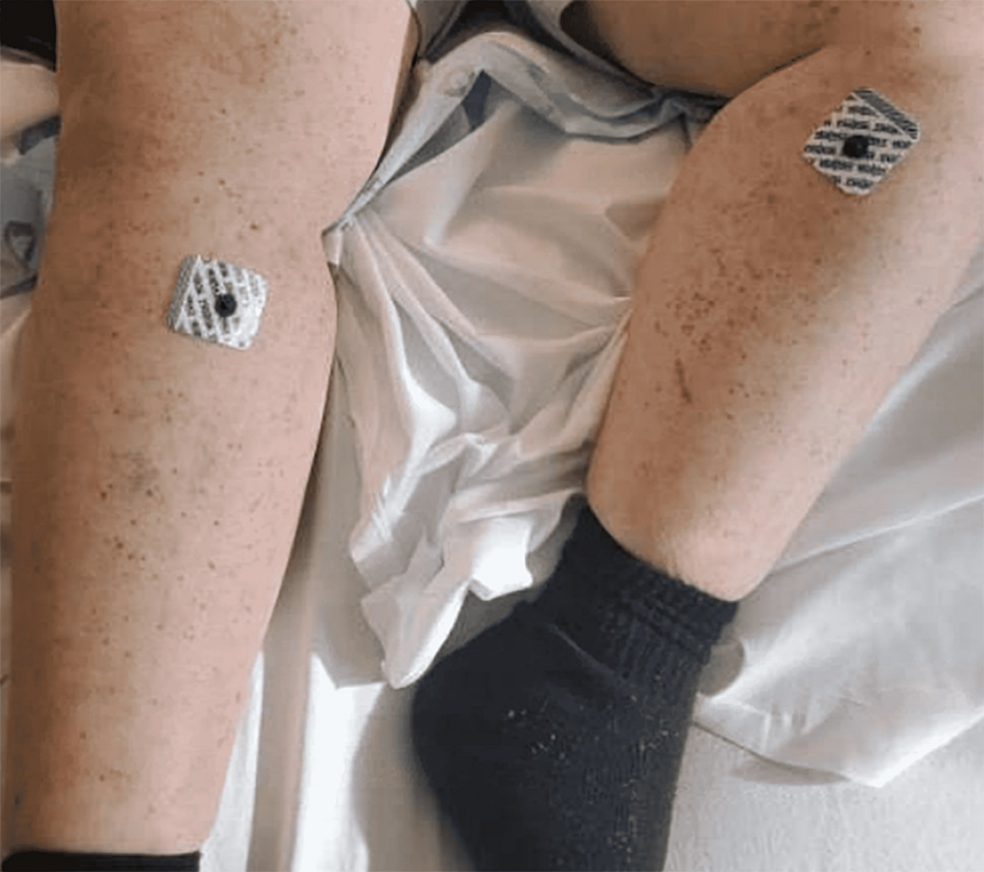
Photograph of patient’s bilateral lower extremities, demonstrating substantial resolution of the patient’s lower extremity rash several days into her treatment.

Rheumatology was consulted and expressed concern for IgA vasculitis. Due to previously noted poor oral tolerance, the patient was started on methylprednisolone 500 mg IV daily. After three days of treatment, the rash, nausea, and swelling began to resolve (Figure 6). She was then transitioned to oral prednisone 60 mg daily, which was prescribed for one week with planned outpatient rheumatology follow-up. Diagnosis of IgA vasculitis was subsequently confirmed via results from a skin biopsy taken during her hospital stay.

## Discussion

This case represents a rare example of adult-onset idiopathic IgA vasculitis, presenting with a unique rash distribution pattern and time course. This patient’s rash was initially limited to the lower extremity and sun-exposed regions, then ascended to involve the abdomen and bilateral upper extremities. The rash of IgA vasculitis tends to be found on the lower extremities, possibly due to gravity slowing blood flow and allowing for increased IgA deposition [3,13]. However, our patient’s rash distribution was atypical, ostensibly appearing to be sunlight-dependent. Her rash first developed in areas that received the most sunlight (her exposed legs and arms), then progressed to areas covered by her clothing (her feet, abdomen, and back). Her sock line, which was the transition point of sunlight exposure on her legs, was sharply demarcated by her rash (Figure 1). Although her feet are the most gravity-dependent parts of her body, they developed a rash days later in her time course. UV-light-dependent IgA purpura has been noted in at least one other case in the literature [17]. Our patient’s rash may also have been exacerbated by the UV rays in sunlight, implying that mechanical damage may play a role in the yet-to-be-clarified pathophysiology of IgA vasculitis rash.

Our patient’s collection of symptoms was also unusual. The literature suggests that adult IgA vasculitis patients typically have musculoskeletal (>70%) and renal (>70%) involvement, with fever (~20%) and nausea or vomiting (~19%) being less common [9]. It is also unusual for GI symptoms to be present at the onset of the disease [9]. In contrast, the presenting symptoms of our patient were severe vomiting and diarrhea, with gastroenteritis, duodenitis, and esophagitis confirmed by esophagogastroduodenoscopy (EGD) and colonoscopy. She denied any arthralgia, and her renal function was unremarkable during her admission, although she subsequently developed renal signs, including proteinuria later in the course of her disease, after discharge.

Our case also highlights the role of high-dose steroids in the management of GI symptoms in adult-onset IgA vasculitis. Although corticosteroids are an efficacious treatment for GI symptoms, their use is somewhat controversial for IgA vasculitis nephropathy [14]. It is also worth noting that corticosteroids may present GI symptoms (e.g., cramping, bloating) as inherent side effects of the medication. Several pediatric studies show no significant change in IgA renal involvement with corticosteroids, nor the prevention of renal symptom development [13]. However, adult IgA vasculitis has higher rates of renal involvement and recurrence, which contribute to its worse prognosis compared to pediatric IgA vasculitis [4]. Although diffuse skin involvement and lack of necrosis are predictive of worsened GI and renal involvement, our patient’s renal function remained stable throughout her hospital stay [12]. Because our patient was unable to tolerate oral medications, she was treated with high-dose IV methylprednisolone early in her hospital stay. This may have resulted in the additional benefit of sparing her kidneys during this IgA vasculitis flare.

It is difficult to conclusively determine how significantly the corticosteroids contributed to the resolution in this case; previous cases with similar presentations have demonstrated resolution with supportive care only, within a few days [18]. The choice to move forward with steroids was particularly difficult in this case, as there were concerns of underlying abdominal infection and steroid exacerbations of abdominal ulcerations. However, the rapid response of the patient to her corticosteroid treatment, and her relative lack of renal impairment, supported the aggressive and early high-dose corticosteroid treatment of adult IgA vasculitis with significant GI involvement.

## Conclusions

Adult-onset IgA vasculitis is relatively rare and typically presents with renal and joint involvement. Presentation with primarily abdominal pain and GI involvement is rarer. A skin biopsy can be used to confirm the diagnosis. High-dose corticosteroid therapy is a treatment option for acute flares.
